# Expanding Understanding of Electrocochleography in Cochlear Implantation: Auditory Neuropathy Spectrum Disorder With Normal Pure Tone Average

**DOI:** 10.1097/ONO.0000000000000035

**Published:** 2023-06-15

**Authors:** Anna C. Buhle, Hilary C. McCrary, Steven A. Gordon, Kathryn M. Johnson, Eric E. Babajanian, Neil S. Patel

**Affiliations:** 1 Virginia Tech Carilion School of Medicine, Roanoke, VA; 2 Department of Surgery, Division of Otolaryngology, University of Utah, Salt Lake City, UT.

**Keywords:** Auditory neuropathy spectrum disorder, Cochlear implant

## Abstract

**Objective:**

Describe the preoperative decision-making, intraoperative electrocochleographic (ECoG) findings, and outcome of cochlear implantation (CI) in a patient with auditory neuropathy spectrum disorder (ANSD) and normal pure-tone thresholds.

**Patients:**

A 19-year-old with a history of hypoxic ischemic encephalopathy and seizures was referred for hearing rehabilitation in the setting of typical hearing by pure tone audiometry but poor speech understanding. A diagnosis of ANSD was made based on acoustic brainstem response (ABR), distortion product otoacoustic emission, and acoustic reflex testing. Imaging revealed no central cause of hearing impairment.

**Interventions:**

Right-sided CI.

**Main Outcome Measures:**

Preoperative and postoperative audiometric data. Intraoperative ECoG.

**Results:**

Preoperatively the patient underwent comprehensive audiologic testing with behavioral audiometry, ABR testing, and CI candidacy evaluation. In the right ear, the pure tone average (PTA) was 15 dB and word recognition score was 36%. ABR confirmed ANSD. Preoperative CNC and AzBio in quiet were 8% and 0%, respectively. Intraoperative ECoG amplitudes and audiometry showed responses in the 100 uV range and estimated PTA of 42 dB HL. Postoperative testing at 1-month post-initial activation revealed PTA of 45 dB HL and unchanged word and sentence scores. However, the patient cites an improved ability to communicate and increased confidence and averages over 14 hours of device use daily.

**Conclusions:**

To our knowledge, this is the first reported case of CI in an ear with normal PTA. Given that nearly all presently available ECoG data comes from patients with greater degrees of hearing loss, this unique case adds to our understanding of hearing preservation in CI.

Auditory neuropathy spectrum disorder (ANSD) is a heterogeneous disorder characterized by normal cochlear function and impaired auditory neural transmission (1–3). Dysfunction may occur at the level of the inner hair cell synapse or along the auditory nerve itself (1–3). Importantly, outer hair cell function remains intact in these individuals; thus, otoacoustic emissions and cochlear microphonics are normal (1–3). Testing should include both psychophysical and physiologic testing such as acoustic brainstem responses (ABRs), otoacoustic emissions, and electrocochleography (ECoG) (2).

Clinically, individuals with ANSD have difficulty with sound perception and localization out of proportion to what is expected based on their pure tone average (PTA) (1,2). This manifests as difficulty understanding speech despite being able to detect it (2). As treatment options have evolved, cochlear implantations (CIs) have emerged as a topic of considerable interest and have been studied with variable results (2–8). This is likely due to the varying locations of the lesion along the auditory system in ANSD; lesions that are pre-synaptic are bypassed by CI and lesions that are post-synaptic may have a mixed response to intervention (1,3,7). CI is thought to “resynchronize” the disorganized neural transmission that characterizes ANSD.

Traditionally, CI is indicated for children with severe to profound hearing impairment and adults with moderate to profound hearing impairment with poor speech understanding (9). Thus, this intervention is generally only considered for patients with ANSD who are not making auditory progress with amplification alone due to impaired sound clarity (speech understanding). Since patients may have severe hearing impairment but also have normal to near normal hearing levels on audiometry, a significant subset of these patients are not considered for treatment with CI. Described herein is a patient with ANSD and normal PTA who was treated with CI. This report describes the preoperative decision-making, ECoG findings, and qualitative and quantitative outcomes to help better understand ANSD and counsel future patients with similar preoperative findings.

## MATERIALS AND METHODS

A 19-year-old male with a past medical history significant for hypoxic ischemic encephalopathy (HIE) following a neonatal intraventricular hemorrhage and seizures presented to neurotology clinic with hearing loss. He did not undergo genetic testing. Throughout childhood, the patient noted multiple normal hearing screenings. More recently, he described worsening hearing, impaired understanding of others, and near-constant nonpulsatile, high-pitched, right-sided tinnitus. He denied other otologic symptoms, including vertigo, otalgia, or otorrhea. There was no family history of hearing loss. A behavioral audiogram demonstrated normal hearing threshold bilaterally, but extremely poor word understanding. An ABR demonstrated findings consistent with ANSD. Given the patient’s history of seizures, an MRI was obtained that revealed no evidence of lesion along the auditory pathways and normal appearing cochlear nerves. He was found to have absent ABR and ipsilateral acoustic reflexes, with present cochlear microphonic and distortion product otoacoustic emissions (Table 1). The latency of the cochlear microphonic was greater than 2.5 msec and the amplitude close to 0.4 uV, which combined were highly suspicious for ANSD. He had bilateral type A tympanograms. Given that he putatively had normal hearing during childhood, it remains unclear whether HIE contributed to the development of ANSD in his case. Nevertheless, it remains possible that the history of HIE and his epilepsy and ANSD are associated in some way.

**TABLE 1.** SPL of the DPOAE amplitudes at specific F2 frequencies

F2 stimulus frequency (Hz)	Right ear DPOAE level (dB SPL)	Left ear DPOAE level (dB SPL)
750	4.1	15.61
1000	7.56	21.78
1500	11.49 (present but abnormal, DP < 0 dB)	23.21
2000	15.46	24.35
3000	17.49	19
4000	14.89	20.86
6000	16.57	30.43
8000	16.82	27.24

DP indicates distortion product; DPOAE, distortion product otoacoustic emission; SPL, sound pressure level.

Given his poor word understanding, a CI evaluation was performed, which revealed AzBio in quiet of 0% and 36% for the right and left ear, respectively (Table 2). The patient and his family were counseled on the unpredictability of CI outcomes in patients with ANSD and the guarded optimism to improve speech understanding and tinnitus. He subsequently proceeded to the operating room for a right CI, as this was his poorer ear on CI testing. Intraoperative ECoG monitoring was completed and an Advanced Bionics HiRes Ultra3D HiFocus device with SlimJ electrode was placed.

**TABLE 2.** CNC and AzBio performance preoperatively versus postoperatively

Feature	CNC	AzBio quiet	AzBio +10 SNR
Preoperative			
Left ear	28% W 49% P	36%	DNT
Right ear	8% W 12% P	0%	DNT
Bilateral	DNT	57%	2%
Postoperative			
Cochlear implant only masked at 60 dB	8% W 20% P	0%	DNT
Cochlear implant + LE	DNT	51%	DNT
Unaided	DNT	50%	DNT

CNC indicates consonant-nucleus-consonant; DNT, did not test; LE, left ear; P, phonemes; SNR, signal-to-noise ratio; W, words.

## RESULTS

**FIG. 1.**
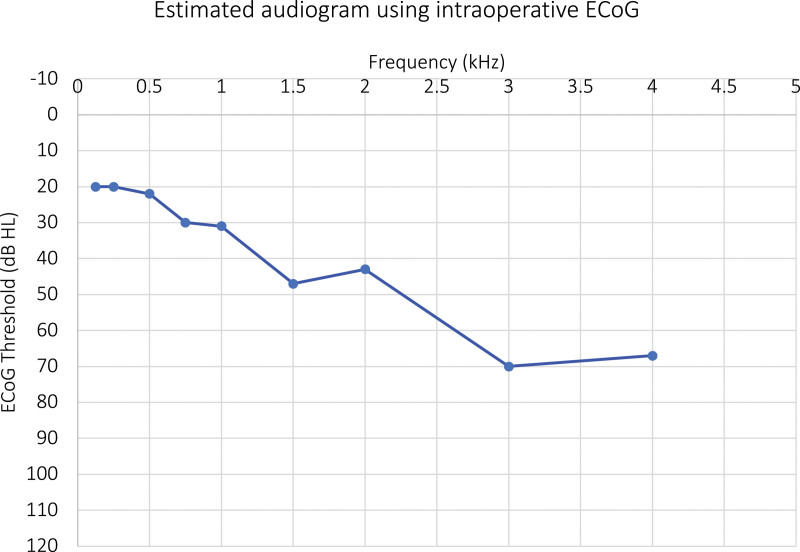
Estimated audiogram using intraoperative ECoG (the values are closely approximated to the software output). Acoustic level (dB HL at 500 Hz) is 75 dB HL. ECoG indicates electrocochleography.

ECoG demonstrated final amplitudes at 500 Hz of 70–90 uV. There was one extracochlear electrode, which was intentional to avoid iatrogenic loss of acoustic hearing, based on ECoG guidance (Fig. 1 and Supplemental Figures 1 and 2, http://links.lww.com/ONO/A17).

**FIG. 2.**
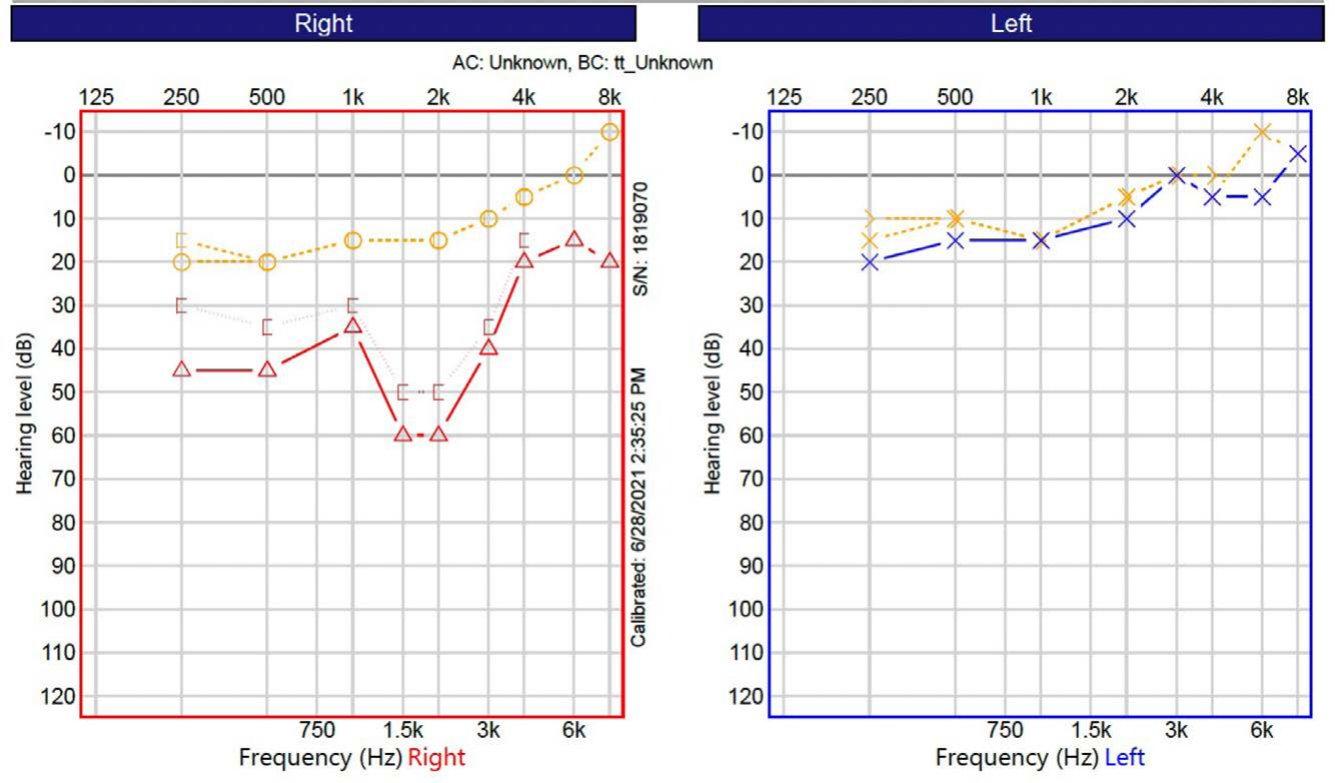
Audiogram demonstrating preoperative thresholds (in yellow) and then postoperative evaluation in red (right) and blue (left). AC indicates air conduction; BC, bone conduction.

The initial activation was completed 1 week after surgery. Two weeks after surgery the patient reported near-constant CI use, with data logging indicated 15.5 hours per day. Subjectively, he felt his hearing and ability to understand others was improved, but voices overall sounded robotic. One month after surgery, a CI assisted audiogram did not demonstrate any improvements from preoperative testing. His AzBio Q remained at 0% (Table 2). However, he continues to have constant use of his CI and subjective improvement. At most recent follow up (approximately 5 months postoperatively), his AzBio sentence score in quiet was 6%. His postoperative audiogram demonstrated similar findings to his intraoperative ECoG including moderate to moderately severe sensorineural hearing loss (Fig. 2). In spite of seemingly poor speech understanding test results, the patient can communicate orally without lip reading with electric only stimulation.

## DISCUSSION

ANSD can manifest as significant hearing loss with preserved PTA, complicating decisions around treatment for these patients. Reported here is a patient with ANSD and normal PTA treated with right-sided CI. The preoperative decision-making, intraoperative ECoG findings, and outcomes of CI in this patient are described here to provide rationale and guidance for providers faced with similar scenarios in their practice.

Numerous studies have investigated CI as a potential treatment for ANSD with variable outcomes (2–8). Berlin et al (10) found that out of 49 patients with ANSD, 85% had successful outcomes with CI. Conversely, Miyamoto et al (4) found that post-CI outcomes in a child with ANSD were worse when compared with children with other causes of sensorineural hearing loss. Currently, CI is considered for patients with ANSD and significant perceptual difficulty who have failed treatment with hearing aids (11). Importantly, CI is generally only considered when patients have a moderate to profound hearing loss on audiometry with poor word understanding (9). Since ANSD is notable for causing loss of speech clarity disproportionate to pure-tone thresholds, an individual may have significant hearing impairment despite having a normal PTA.

The literature on treating patients with ANSD and normal to near-normal PTA is sparse. A case series from Santarelli et al (12) describes CI in an 18-year-old female with auditory neuropathy and mild right-sided hearing loss (PTA 28 dB on the right side), with an improvement in open-set speech recognition post-implant. Dean et al (13) report 3 patients with normal preoperative PTA who were implanted; however, the preoperative and intraoperative decision-making and post-implant outcomes for these patients are not specifically reported.

The patient described herein experienced a significant subjective improvement in his hearing as evidenced by high daily use and reported satisfaction. However, his pre- and post-implant objective testing was unchanged. Audiometric and ECoG findings suggest that the lesion is likely post-synaptic, which may explain the unchanged objective postoperative results (10). However, the reason for conflicting subjective and objective results found in this patient is not clear. Alternative means of assessing functional improvement are vital in nontraditional cases such as these. Furthermore, the correlation between speech recognition ability and Cochlear Implant Quality of Life responses is weak, suggesting that other unmeasured factors are impacting the quality of life of CI users (14). Quality of life surveys and disease-specific instruments intended to assess the functional impact of hearing loss would have been useful in outcome assessment.

Our patient underwent extensive preoperative counseling before a decision was made to proceed with CI. Counseling is particularly necessary due to variable success rates across individuals with ANSD. This is due to several factors including differing loci of lesions and genetic mutations, different types of CI used, and diverse patient factors such as age, socioeconomic status, and comorbid developmental delay (3,15). The patient’s desire to gain hearing ability must also be assessed, as some patients may be comfortable with their identity as hearing-impaired. We recommend in-depth counseling for any patient with ANSD being considered for CI.

Although results have been mixed, ECoG remains the most promising intraoperative tool to optimize short-term hearing preservation. In this case, postoperative 4-frequency PTA was 37.5 dB HL with a rising configuration. Notably, high frequency thresholds were nearly unchanged from preoperative levels, and a small air-bone gap was present in the low to mid frequency range. There are several questions generated by these findings. First, what are the true maxima of hearing preservation at each threshold? This question follows from the fact that high frequency thresholds at the base of the cochlea are the most “preserved” in this case. Is the resonance of the ossicular chain or dynamics of the organ of Corti affected by having an electrode in the scala tympani? Perhaps the relative size of the scala compared to the electrode volume plays a role. How do we interpret residual hearing preservation when pure-tone thresholds are normal in the high-frequency range (in contrast to most hearing preservation CI cases where the only measurable thresholds are in the low- to mid-frequency range)?

In conclusion, ANSD is a difficult to treat cause of hearing impairment that varies across individuals in both clinical presentation and severity. Many patients may have hearing levels ranging from normal to mild hearing impairment but experience significant difficulty in speech discrimination. Presented here was a patient with ANSD and normal PTA who experienced subjective improvement in his ability to hear and understand following CI. Near complete hearing preservation was achieved in the high frequency range, which has not been described given the typical hearing pattern of patients undergoing CI with residual hearing. Subjective outcome measures should be used in these cases to assess functional changes in these complex patients.

## FUNDING SOURCES

None declared.

## CONFLICT OF INTEREST

None declared.

## DATA AVAILABILITY STATEMENT

Data sharing is not applicable to this article as no datasets were generated or analyzed during the current study.

## Supplementary Material


