# Supplementary material for: Expanding Understanding of Electrocochleography in Cochlear Implantation: Auditory Neuropathy Spectrum Disorder With Normal Pure Tone Average
Source: Otol Neurotol Open. 2023 Jun 15;3(2):e035. doi: 10.1097/ONO.0000000000000035 (PMC10950127; doi:10.1097/ONO.0000000000000035)
Supplement: Supplementary file 1 [file ono-3-e035-s001.pdf]

Electrode Sweep

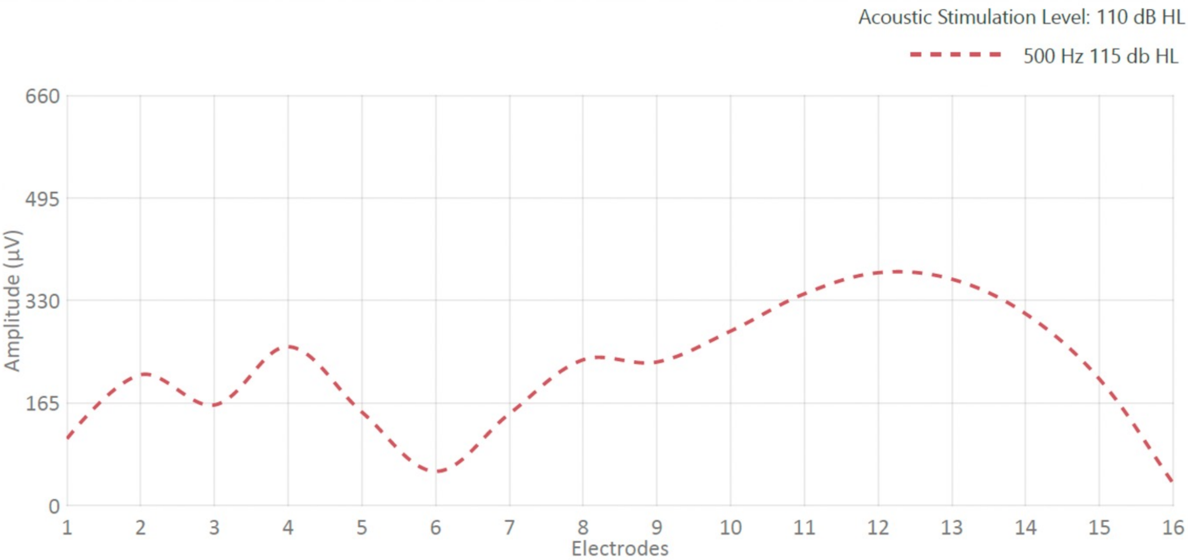

Impedances

Electrode	1	2	3	4	5	6	7	8	9	10	11	12	13	14	15	16
Impedance (kΩ)	2.8	2.7	2.3	2.6	2.7	2.6	2.7	2.4	2.4	2.3	2.8	2.8	3.2	3.9	3.1	4.6

Valid
